# Lower pattern recognition memory scores in anorexia nervosa

**DOI:** 10.1186/s40337-021-00406-8

**Published:** 2021-04-17

**Authors:** Johanna Keeler, Ellen Lambert, Miriam Olivola, Judith Owen, Jingjing Xia, Sandrine Thuret, Hubertus Himmerich, Valentina Cardi, Janet Treasure

**Affiliations:** 1 Section of Eating Disorders, Institute of Psychiatry, Psychology and Neuroscience, King’s College London, 103 Denmark Hill, London, SE5 8AF UK; 2 Department of Mental Health and Addictions, Azienda Socio-Sanitaria Territoriale di Pavia, Pavia, Italy; 3 Department of Basic and Clinical Neuroscience, Institute of Psychiatry, Psychology and Neuroscience, King’s College London, London, UK; 4 Department of Neurology, University Hospital Carl Gustav Carus, Technische Universität Dresden, Dresden, Germany; 5 Department of General Psychology, University of Padova, Padova, Italy

**Keywords:** Anorexia nervosa, Pattern separation, Recognition memory, Mnemonic similarity task, Hippocampus

## Abstract

**Background:**

There is extensive evidence for volumetric reductions in the hippocampus in patients with anorexia nervosa (AN), however the impact on function is unclear. Pattern separation and recognition are hippocampus-dependent forms of learning thought to underlie stimulus discrimination.

**Methods:**

The present study used the Mnemonic Similarity Task to investigate pattern separation and recognition for the first time in patients with AN (*N* = 46) and healthy controls (*N* = 56). An Analysis of Covariance examined between-group differences, controlling for age, antidepressant use and method of task delivery (remote vs. in person).

**Results:**

When controlling for covariates, pattern recognition memory scores were lower in the AN group with a medium effect size (*d* = 0.51). In contrast, there was a small effect whereby patients with AN had a greater pattern separation score than controls (*d* = 0.34), albeit this difference was not significant at the *p* = 0.05 threshold (*p* = 0.133). Furthermore, pattern separation and recognition memory abilities were not related to age, body mass index, eating disorder psychopathology or trait anxiety levels.

**Conclusions:**

This preliminary study provides initial evidence for an imbalance in pattern separation and recognition abilities in AN, a hippocampus-dependent cognitive ability. Further studies should endeavour to investigate pattern separation and recognition performance further in AN, as well as investigate other hippocampus-dependent functions.

**Supplementary Information:**

The online version contains supplementary material available at 10.1186/s40337-021-00406-8.

## Plain English summary

The hippocampus is an area of the brain that is vital for memory and learning, and it is not understood the extent to which its function is impaired in anorexia nervosa (AN). This paper used the Mnemonic Similarity Task to assess pattern separation, a hippocampus-dependent form of memory, in AN. This task involves showing participants a sequence of objects, who then categorise them as “indoor” or “outdoor” objects. Participants are later shown a sequence of objects, although some of the images are replaced by a similar but slightly different image. The task involves recognising whether an image has previously been seen (pattern recognition) and also whether it is similar to, but distinct, from a previous image (i.e. pattern separation). In this study, individuals with AN showed reduced performance in pattern recognition, when statistically controlling for their age, how the task was delivered and their use of antidepressant medication. However, their performance in pattern separation was intact. This may indicate an imbalance in this hippocampus-dependent form of memory in AN.

**Supplementary Information:**

The online version contains supplementary material available at 10.1186/s40337-021-00406-8.

## Introduction

Anorexia nervosa (AN) is a serious, complex psychiatric illness, with core characteristics including an avoidance of food, leading to severe dietary restriction and a significantly low body weight [1]. Many patients with AN have an enduring illness, with one study finding only a third recovered at a 9-year follow-up [2]. The protracted starvation impacts adversely on brain function and volume and the chronic state can be treatment resistant and often comorbid with depression (see the Cognitive Interpersonal Model [3];. Treatment resistance in other forms of severe psychiatric illnesses such as Major Depressive Disorder and schizophrenia has been linked to abnormal hippocampal function [4, 5]. In a scoping review, preliminary evidence was found for a reduction in the hippocampal structure in AN [6]. However, there were very few studies that examined hippocampal function in AN.

Adult hippocampal neurogenesis (AHN) is thought to be essential for specific forms of memory and learning processes [7]. AHN pertains to the birth of new neurons from stem cells in the granule layer of the dentate gyrus [8]. Reductions in AHN are associated with reductions in reversal learning and cognitive flexibility [9], both of which are impaired in AN [10, 11]. Reduced AHN in AN has been hypothesised due to aberrations in inflammatory molecules such as brain-derived neurotrophic factor and vascular-endothelial growth factor, which leads to secondary changes in affect and cognition, similar to proposed mechanisms within the depression literature [12–14]. Moreover, evidence from studies on rodents suggests that chronic mild stress [15] and starvation [16] reduces both AHN and hippocampus-dependent forms of learning. Both chronic stress (physiological and psychological) and chronic starvation are features of AN. Concurrently, animal models of AN have indicated preliminary evidence for abnormalities in hippocampal proliferation, which is the initial process towards AHN [17]. These findings raise the possibility that other neurogenesis-dependent functions of the hippocampus that have not yet been investigated, such as pattern separation and recognition, may be impacted in AN.

Pattern separation pertains to the process in which overlapping or similar representations are transformed into separate memory engrams, a crucial aspect of episodic memory. This allows the hippocampus to distinguish between highly similar but distinct representations at the time of encoding and storage. Pattern completion occurs at the time of retrieval, where the hippocampus reinstates the entire stored pattern in the case of a partial or degraded retrieval cue – rather than creating a new memory representation. Intact pattern separation at the time of encoding is vital to ensure distinct memory patterns are stored for their later retrieval. As such, interference after initial pattern separation can affect later recognition performance [18]. There is debate on whether pattern separation and completion are two ends of a unitary process, or whether they are distinct processes reliant on different neural networks [19, 20]. Indeed, individuals with hippocampal damage can be impaired in pattern separation but have preserved recognition performance [21]. The same pattern is often observed in age-related decline [22, 23]. As such, it is now generally thought that pattern separation and completion are distinct processes that are at odds with one another [24].

Age-related decreases in pattern separation have been associated with volumetric reductions in the dentate gyrus subfield of the hippocampus [25]. On the other hand, the cornu ammonis (CA) 3 subfield is thought to be a vital component of pattern recognition performance, which volumetrically is age-independent [25]. People with AN have been shown to have specific reductions in these subfields when controlling for total brain volume [26]. The processes of pattern separation and recognition have been linked to AHN, although there is a paucity of human evidence due to difficulties in directly measuring neurogenesis. However, this is possible in animal models. Cellular studies in rodents indicate that intact pattern separation is thought to link to “new” granule cell neurogenesis in the dentate gyrus, and pattern recognition is facilitated when these newly born cells are integrated into existing networks [27]. Specifically, “old” granule cells in the dentate gyrus may mediate the rapid retrieval of memory engrams from the CA3, thus facilitating pattern completion (i.e. pattern recognition). In many human fMRI studies, the CA3 and dentate gyrus are difficult to distinguish and thus signals are combined to reflect pattern separation, whereas cornu ammonis 1 (CA1) activity, which receives input from the CA3, is thought to reflect pattern completion [28].

Clinically, impaired pattern separation can lead to excessive overgeneralisation, whereby an individual perceives multiple stimuli as similar, even though they are dissimilar [29]. Pattern separation and completion impairments are particularly pronounced in mild cognitive impairment [30] and neurodegenerative diseases such as Alzheimer’s disease [22]. Impairments in neurogenesis and hippocampal functioning are thought to be of relevance to a range of psychiatric and developmental disorders. For example, there is empirical evidence for pattern separation disturbances in schizophrenia [31], autism spectrum disorder [32] and depression and anxiety-related psychopathology [33–35]. The transdiagnostic features of psychosocial stress and sleep disturbances may contribute to these problems [36–39].

In animals, calorie restriction has been associated with changes in hippocampal functioning [40–42]. The negative effects of caloric restriction on hippocampal neurogenesis in rodents are particularly pronounced during adolescence [16]. An intermittent calorie-restricted diet used for the treatment of obesity in humans was associated with an increase in pattern separation performance, but a reduction in pattern recognition [43]. However, increases in pattern recognition performance have been found by promoting mastication through prescribing the daily chewing of gum [44]. Furthermore, exercise, especially in adolescents, also increases neurogenesis in animal models [45, 46]. Exercise in human studies has also been shown to increase pattern separation [47]. In summary, whilst caloric restriction and exercise may improve pattern separation, recognition abilities are impaired by restriction and promoted by high levels of mastication. Given that AN is characterised by dietary restriction (and thus lower mastication) and often compulsive exercise, it may be expected that pattern recognition abilities are impaired and pattern separation abilities are increased in this population.

Antidepressant usage has also been found to increase neurogenesis in both human and animal studies. The mechanism is thought to be by reducing hypothalamic-pituitary-adrenal (HPA) axis overactivation and cortisol release and increasing brain-derived neurotrophic factor (BDNF), which plays an important role in nerve growth and development [48]. Indeed, certain antidepressants have also been found to increase neurogenesis in both human and animal studies, and their ability to do so has been deemed relevant for the successful treatment of the psychiatric disorders they are used for [49]. Therefore, we considered the use of antidepressant an important potential confounding factor influencing neurogenesis- and neuroplasticity-dependent hippocampal functions. There has been little examination of the behavioural effect of antidepressants on pattern separation and recognition abilities in the literature, although it has been suggested as a key avenue for future research (e.g. [50, 51]).

The present experimental study is to our knowledge the first investigation of pattern separation and recognition abilities in AN. From the literature we predicted that people with AN would show a reduction in pattern recognition with an increase in pattern separation. Moreover, we predicted that pattern separation and recognition memory performance would be confounded by variables such as antidepressant usage and age. Finally, we sought to examine the extent to which pattern separation and recognition abilities were related to clinical features such as trait anxiety, illness severity and weight.

## Methods

### Participants

The clinical group were recruited from the South London and Maudsley NHS trust and from recruitment websites, such as the eating disorder charity BEAT website (www.beateatingdisorders.org.uk). The healthy control group was recruited through the University internal recruitment circular email sent to staff and students. The clinical group included individuals aged between 18 and 60 with a formal or self-reported diagnosis of AN at varying stages of treatment and weight restoration. The healthy control (HC) group included individuals with no history of any mental health condition, including eating disorders, and were not taking psychiatric medication at the time of the study (ages between 18 and 61).

A total of 102 participants took part in this study (46 in the clinical group, 56 in the healthy control group). Previous studies using the mnemonic similarity task in novel populations have found clinically relevant effects with group sizes of 20–30 [30, 43, 52]. A compromise power analysis was conducted using G*Power software in order to assess the statistical power of this study, using effect sizes of varying sizes. Based on a small effect size of *d* = 0.2, a sample of 85 participants (the sample used in the pattern separation main analyses) entered into an ANCOVA model with three covariates yielded an estimated power of 0.58. With a moderate effect size of *d* = 0.5, the same model yielded an estimated power of 0.83.

Informed consent was obtained from all individuals before study participation and participants were reimbursed with a £5 voucher.

### Procedure

Participants completed the pattern separation task before the self-report questionnaires. The Mnemonic Similarity Task was used (MST [30];). Participants completed the task and questionnaires either on a study computer with the researcher present (*N* = 55; AN = 24, HC = 31) or on a personal computer with a stable internet connection (*N* = 47; AN = 22, HC = 25), via a remote version of the task delivered using Inquisit technology [53]. This was due to the COVID-19 pandemic interrupting the ability to conduct face-to-face research.

### Measures

*Self-report questionnaires*: participants provided information in relation to their age, height, weight, current use of psychiatric medication and co-morbidities, ethnicity, clinical treatment status, living status (e.g. living with parents, in own accommodation), and level of educational attainment. We also asked participants questions based on their average sleep duration, specifically “on average how many hours of sleep do you think you get a night?”. The Eating Disorder Examination Questionnaire (EDE-Q [54];) was included to assess current ED-related symptomology. The trait subscale of the State-Trait Anxiety Inventory (STAI-T [55];) was included to assess trait anxiety.

*Pattern separation task*: Participants were given standardised instructions for the task at the beginning of the session, which explained the stages of the task, identified the response keys and finally gave the opportunity for questions. Participants completing the task in person were read instructions from a script, whereas participants administered the task remotely read an identical script. For this latter group of participants, instructions in the second part of the task (recognition phase) were read aloud by an automated male voice. These participants were also not accompanied when completing the task, although they were encouraged to contact a researcher who was available at the time of testing, if they had any questions. Timings of the task are standardised by the Stark Laboratory, facilitating comparisons across datasets. During the initial encoding phase, participants viewed 128 object images on a computer screen. The images were colour photographs of everyday objects on a white background (see [30] for more details). Participants were instructed to classify the images as either “indoor” or “outdoor” objects (based on their opinion), by pressing specific buttons on their keyboard. Participants receiving the task remotely were given practice trials, in order to demonstrate the task procedure, albeit due to technological limitations this was not possible in person. Each image was presented for 2 s, and the inter-stimulus interval was 0.5 s. The encoding phase lasted for 5.3 min in total. After a delay of several minutes, a retrieval phase began. During the retrieval phase, participants viewed 192 object images; one third (*n* = 64) of which were completely new images (foils) one third of which were identical to the images presented in the encoding phase (targets or repeats), and a final third which were similar to the images presented during the encoding phase (lures). Foil, target and lure stimuli were presented randomly. Participants were instructed to classify these images as “new”, “old” or “similar items”, by pressing specific buttons on their keyboard. Each image was presented for 2 s, and the inter-stimulus interval was 0.5 s. The retrieval phase lasted for 8 min in total. All participants were presented with object stimuli from Sets C or D [30].

As in previous studies (e.g. [30]), the Lure Discrimination Index (LDI) is the main outcome measure. LDI was calculated as the difference between the rate of “similar” responses given to the lure items minus the rate of “similar” responses given to the foil items. A higher LDI reflects better behavioural pattern separation performance. The traditional recognition memory score for repeat items was calculated as the difference between the rate of “old” responses given to repeat items minus the rate of “old” responses given to the foil items. A higher recognition score reflects better general recognition abilities.

### Data processing and analysis

Chi-squared tests and *t*-tests were used to compare demographic characteristics, EDE-Q scores and STAI-T scores between groups. Bonferroni corrections were applied to correct for multiple comparisons on subscales of the EDE-Q, with a significance level *p* of (0.05/4) = 0.0125.

Pattern separation and recognition memory scores were compared using an Analysis of Covariance (ANCOVA), in order to control for the effects of age, method of task delivery and antidepressant use. Age and antidepressant usage were selected as covariates due to the aforementioned findings that pattern separation abilities are known to decline over the lifespan and can theoretically be affected by the use of drugs such as selective serotonin reuptake inhibitors (SSRIs), which increase AHN [51]. Given the differences in procedure between the remote and in-person versions of the MST, method of task delivery was also included as a covariate. Relationships between variables (eating disorder psychopathology, trait anxiety, age, pattern separation and recognition memory scores) per group were assessed using Pearson product-moment correlation coefficients®.

For all analyses, effect sizes were established using Cohen’s d and interpreted as small (0.2), medium (0.5) and large (0.8) [56]. A *p*-value of <.05 was interpreted to warrant further investigation. Analyses were conducted in SPSS version 25 (SPSS, Inc., USA, IBM SPSS Statistics for Windows, Version 25.0. Armonk, NY: IBM Corp.). A statistician at King’s College London was consulted during the analysis process.

#### Process for dealing with missing data

Twelve participants did not contribute data to a selection of demographic and clinical variables, some of which were used as a covariate (e.g. antidepressant usage). These variables included: living status, level of education, relationship status, ethnicity, psychiatric comorbidities, medication usage, average sleep, STAI-T, EDE-Q Global Score and all subscales of the EDE-Q. The percentage of missing values varied between 11.7 and 18.4%, with a total of 298 out of 1247 records of these variables (19.3%) being incomplete. Supplementary Table 1 describes the missing data rates of each variable (see Additional file 1).

A Little’s missing completely at random test of data patterns revealed that this missingness was not completely at random (MNAR). Furthermore, separate variance t-tests revealed that missing values were significantly correlated with values of one or more variables in the dataset, indicating missingness at random (MAR). Thus, we performed multiple imputation, using the regression method in SPSS. Five imputed datasets were performed, which were pooled for analyses. All missing variables were also included in the imputation model as predictors, and age was added as an auxiliary variable.

Pooled data using multiple imputation is used in the analysis of demographic and clinical characteristics, as well as in the Pearson product-moment correlations. Due to software limitations, our main analyses could not be performed on the pooled imputed data. As such, we primarily report the ANCOVA models for complete-case analyses. For comparison, we also performed the analysis on each imputed model separately.

## Results

### Clinical and demographic characteristics

Table 1 describes the clinical and demographic characteristics of the participants in the control and clinical groups after multiple imputation. Differences in demographic and clinical characteristics were identical when comparing the imputed dataset and the complete-case dataset.

**Table 1:** Clinical characteristics and memory scores of the participants by group

	Clinical Group (***n*** = 46)	Healthy Controls (***n*** = 56)	Between-groups difference			
	Mean (***SEM***)	Mean (***SEM***)	t	df	***p***	***d***
Age (Years)	30.43 (*1.44*)	26.63 (*1.09*)	−2.14	100	0.03*	−0.43
Body Mass Index (kg/m^2^)	15.00 (*0.27*)	22.07 (*0.53*)	11.33	100	< 0.001*	2.26
Trait Anxiety (total score)	65.11 (*1.93*)	39.40 (*1.44*)	−11.0	100	< 0.001*	−2.19
EDE-Q Global Score	3.97 (*0.22*)	0.92 (*0.09*)	−13.17	100	< 0.001*	−2.62
EDE-Q Restraint	3.64 (*0.51*)	0.57 (*0.10*)	−6.10	100	< 0.001*	−1.21
EDE-Q Eating Concern	3.08 (*0.26*)	0.36 (*0.06*)	−10.85	100	< 0.001*	−2.16
EDE-Q Shape Concern	4.65 (*0.21*)	1.58 (*0.15*)	−12.46	100	< 0.001*	−2.48
EDE-Q Weight Concern	4.46 (*0.22*)	1.22 (*0.13*)	−12.90	100	< 0.001*	−2.57
Pattern Separation (LDI)	0.33 *(0.03)*	0.29 *(0.03)*	−1.20	100	0.231	−0.24
Recognition Memory	0.74 *(0.02)*	0.75 *(0.02)*	0.75	94	0.451	0.09
	**N (% of group)**	**N (% of group)**	**X**^**2**^ **(*p*-value)**			***d***
Highest level of education			9.18 (0.01)*			0.63
GCSE	**6 (13%)**	3 (5%)				
A-Levels	18 (39%)	10 (18%)				
Higher education	22 (48%)	43 (77%)				
Average sleep duration^†^			30.79 (< 0.001)*			1.32
>8 h	9 (20%)	2 (39%)				
6–7 h	9 (20%)	28 (50%)				
5–6 h	17 (37%)	6 (11%)				
0–5 h	11 (23%)	0 (0%)				
Medication use^†^			44.70 (<.001)*			1.77
Antidepressant	20 (44%)	0 (0%)				
Antipsychotic + Antidepressant	7 (15%)	0 (0%)				

*Note*. *EDE-Q* Eating Disorder Examination Questionnaire, *LDI* Lure Discrimination Index, *SEM* standard error of the mean, *p<0.05, ^†^The pooled test statistic for this categorical variable was calculated using the median value [57]. For measures with multiple subscales, *p*-values which remained significant after applying Bonferroni Correction are displayed in bold

There was one male participant and one participant who identified as non-binary. Participants in the clinical group were older than participants in the control group, were less ethnically diverse (91% AN vs 61% HC being white) and were more educated (see Table 1), but did not differ on any other demographic characteristics such as living status. As expected, participants in the clinical group had a significantly lower body mass index and reported significantly higher levels of eating disorder psychopathology. Participants in the clinical group had significantly different average sleep duration and medication usage.

Within the clinical group, 50% were receiving inpatient treatment, 26% were receiving outpatient treatment and 24% were not receiving any current treatment. Of this group, 24 participants had a confirmed diagnosis of AN (were recruited from within hospitals) and 22 had a self-reported diagnosis of AN. The majority (97%) of participants who reported their weight had a BMI lower than 18.5 kg/m^2^. Furthermore, 32% of participants in the clinical group reported having a comorbid anxiety disorder, 52% reported comorbid depression, 7% a comorbid diagnosis of emotionally unstable personality disorder and 2% “suspected” Autism Spectrum Disorder.

### Mnemonic similarity task performance

There were small differences in LDI scores (pattern separation scores) between groups in the pooled t-test analyses, however effect sizes between groups for recognition memory were negligible (see Table 1 for effect sizes). Response proportions for each stimulus and response type are presented in Supplementary Table 2 (see Additional file 1).

Two individual one-way ANCOVAs were conducted using a complete-case analysis, to control for potential confounders of age, antidepressant use and the method of task delivery (i.e. in person vs. remote administration; see Table 2). In this analysis, medication usage was not recorded for 17 participants (HC = 2; AN = 17) thus these participants were excluded from analyses. Scores from seven participants (HC = 6; AN = 1) were identified as outliers in the recognition memory variable and thus were excluded from analyses in order to meet the assumptions of the statistical test. Levene’s test and normality checks were carried out following this and assumptions were met for each dependent variable.

**Table 2:** Mnemonic Similarity Task between-groups ANCOVA controlling for age, antidepressant use and method of mnemonic similarity task delivery

	Clinical Group	Healthy Controls	Between-groups difference			
	Estimated Marginal Mean (***SD***), N	Estimated Marginal Mean (***SD***), N	F	df	***p***	***d***
Pattern Separation Scores (LDI)	0.36 (*0.22*), 31	0.28 (*0.21*), 54	2.22	1,80	0.133	0.34
Effect of Age			1.28	1,80	0.261	0.26
Effect of Antidepressant Use			1.05	1,80	0.309	0.23
Effect of Method of Delivery			1.05	1,80	0.308	0.23
Recognition Memory Scores	0.69 (*0.14*), 31	0.77 (*0.14*), 48	4.72	1,74	0.033*	0.51
Effect of Age			0.76	1,74	0.385	0.20
Effect of Antidepressant Use			5.17	1,74	0.026*	0.53
Effect of Method of Delivery			1.23	1,74	0.272	0.26

*Note*. *SD* standard deviation, *LDI* Lure Discrimination Index, *p < 0.05

In this first ANCOVA there was no significant effect of diagnosis on pattern separation, however the clinical group had higher scores with a small effect size. A separate ANCOVA showed significant differences in recognition memory scores with lower scores in the clinical group. Furthermore, antidepressant usage emerged as a significant covariate in this model. Within the clinical group, participants who reported antidepressant usage had a higher pattern recognition score (M = 0.77, SD = 0.12) than those who were not taking antidepressant (M = 0.68, SD = 0.13) with a medium effect size (Cohen’s *d* = 0.73).

#### Comparison with pooled multiple imputation analysis

As aforementioned, in other to verify the results of the complete-case ANCOVA, following the imputation of missing data, we performed identical ANCOVAs to above with each of the five imputed datasets. For both ANCOVA models, overall significance and individual covariate significant remained the same across all imputed datasets. This indicates the results are consistent when correcting for missing data.

### Correlation analyses for pattern separation outcomes

Results of the Pearson product-moment correlation coefficients per group are presented in Supplementary Tables 3 and 4 (see Additional file 1). These analyses were based on pooled data from imputed datasets. Pattern Separation scores were positively associated with recognition memory scores (*p* < 0.05) in the clinical group but not in the control group. Pattern separation and recognition memory scores were not associated with age, body mass index, eating disorder symptoms or trait anxiety (*p* > 0.05) in both the clinical and control groups.

## Discussion

The present study was the first investigation of pattern separation and recognition abilities in AN. Recognition memory was significantly lower in the AN group with a medium effect size when controlling for age, medication usage and task delivery. However when using the same covariates, there was a small increase in pattern separation scores albeit this statistically was not significantly different. There was no association of pattern separation or recognition scores with clinical features such as eating disorder psychopathology, BMI and trait anxiety levels.

Our main finding was the lower recognition memory scores specific to the AN group. This profile is similar to the previous findings in people with obesity, who followed a 4-week intermittent calorie restricted diet and showed a reduction in recognition memory [43]. Cellular studies using animal models have indicated that pattern separation is contingent on the birth of “new” adult born granule cells in the dentate gyrus, whereas pattern completion (i.e. recognition) is contingent on the survival and integration of these cells, which become “old” adult born granule cells [27]. Animal models have shown that factors such as dietary restriction reduces the survival of these newly born cells in the ventral dentate gyrus, due to the newly born cells being unstable [42]. This offers a possible explanation for our finding of reduced recognition memory in AN, since dietary restriction is characteristic of the disorder and is detrimental to both the survival of newly born cells in the dentate gyrus and pattern recognition performance. However, it is important to consider the difference of 0.08 (or 8%) in the context of clinical relevance. There are no published normative data for this task, although for a similar age group as in our study [20–39], previous research has indicated an average recognition memory score of approximately 0.80 (80%) in healthy individuals [58], which is generally stable across the lifespan. In populations with mild cognitive impairment (MCI [59];) and Alzheimer’s Disease (AD [60];), recognition scores have been estimated at 0.57 (57%). The corrected recognition score for the AN sample used in this study was 0.69 (69%), which lies between estimates for healthy controls and for serious memory disorders such as MCI and AD. It has been suggested that recognition memory measured by the MST is sensitive to CA3 function in the hippocampus, and thus our findings may be indicative of a subtle CA3 impairment [25]. However, a difference of 8% can be seen as marginal and with limited published data on recognition memory reductions measured by this task, it is difficult to speculate on how this translates to clinical relevance.

Moreover, this study also found that pattern separation and recognition performance was unrelated to clinical features of AN, such as eating disorder psychopathology and BMI. This is incongruent with the literature in depression, whereby depression scores were inversely related to performance on the MST [61]. However, the concept of “neuroprogression” has been recently introduced into the eating disorder literature [3, 62], which pertains to the pathological rewiring of the brain along the course of the illness (see [63] for a detailed overview of the term). This may contribute to impairments in cognitive processes as the illness progresses, partly due to the effects of persistent low weight and dietary restriction on the brain. Therefore, illness duration, as opposed to BMI, may be an important component of neuroprogression to examine in relation to pattern separation and recognition abilities in future research.

Interestingly, these results do not indicate that a tendency to overgeneralise learning exists in AN, as is thought to underlie other psychiatric disorders such as post-traumatic stress disorder [64], depression [65] and anxiety disorder [66]. This is especially pertinent given half of the clinical sample reported comorbid depression. The findings indicate that this sample can actually distinguish between stimuli during the encoding phase well (i.e. separate) but upon retrieval (i.e. completion) are less able to correctly retrieve the distinct representations they had stored. This may indicate an inability to store representations from a previous learning episode and apply this learning later on. Overall, our findings may suggest that an imbalance in this hippocampus-dependent system also exists in AN, albeit different to that observed in depression, which should be investigated further.

Crucially, when controlling for antidepressant usage, previously unseen deficits in pattern recognition performance emerged. Within the clinical group, those taking antidepressant medication also showed a greater pattern recognition performance than those not taking medication. Antidepressants are considered to produce their benefit through increasing hippocampal neurogenesis [9, 67]. Post-mortem studies have shown that individuals with major depressive disorder (MDD) undergoing chronic antidepressant treatment have a larger dentate gyrus than both untreated MDD and healthy controls [68]. They also have greater numbers of neural progenitor cells, which are cells capable of dividing and differentiating, and dividing cells, which become part of the granule layer of the dentate gyrus [68]. Furthermore, anterior hippocampus volume is increased in those with MDD who are receiving antidepressant treatment [69]. As such, it is possible that antidepressant treatment in the clinical sample normalised an imbalance in the hippocampus-dependent system, in this sample.

This study suffers from a number of methodological limitations that should be considered in the interpretation of our results and addressed in further investigation. Firstly, there are key variables known to impact pattern separation abilities that were not measured in this study. We did not have a measure of physical exercise, which has been found to promote pattern separation performance in humans [47]. Thus, the high levels of exercise often seen in AN might be a confounding factor, which should be measured in future studies. Other important factors that were not measured in this study include levels of mastication, which should be addressed in future research through measuring time chewing as well as their current dietary input. Measures of illness or amenorrhea duration, other psychopathologies (e.g. depression and stress) and indices of general cognitive ability (e.g. attention span, general intelligence) were not measured in this study. The AN group was more educated than the HC group in this study, and it is unclear from the literature whether education or IQ are influential on pattern separation and recognition abilities, although education has been found to have an influence on neuropsychological task performance (e.g. [70]). Therefore, these factors may be important confounds or moderators to consider when measuring these specific hippocampal abilities (e.g. [71]). As a result of missing data, a complete-case analysis was used in the primary analyses. However, following a multiple imputation of missing data, results across imputed datasets with a full dataset were consistent with results from the complete-case analyses. Finally, it is possible that the study was underpowered. Post-hoc compromise power analyses were conducted in order to assess the observed statistical power of this study. When assessing an ANCOVA model with three covariates, estimated power based on our observed effect sizes ranged from 0.70 (*d* = 0.34) to 0.82 (*d* = 0.51). Based on these estimations, there is a chance that the detection of effects was limited by our sample size, in the context of the pattern separation analyses. However, when considering these power estimates, it should be emphasised that estimates of power based on observed effects may be analytically misleading and may not reflect true power [72]. Nonetheless, the results from this study will be useful in development of future investigations and for future meta-analyses.

## Conclusion

The present study provides preliminary evidence for the presence of lower pattern recognition memory yet intact pattern separation in individuals with AN. Clinically, individuals with AN often show memory lapses, problems with recalling specific details of episodic memories and difficulties in thinking about the future. Individuals with AN in this study were able to distinguish between details well but were also unable to correctly recall details of the stimuli. These findings may shed some light on their difficulties in thinking about the future and imagining recovery, since this requires the ability to flexibly recombine details from past memories [73]. The inability to imagine a future may act as a barrier to recovery and maintaining hope [74].

Further research would benefit from conducting a larger study in order to replicate these findings as well as distinguishing AN groups based on key variables that have strong effects on hippocampal neurogenesis and pattern separation performance (e.g. level of exercise and mastication) when examining pattern separation performance, in order to address potential confounds.

## Supplementary Information

**Additional file 1.**

## Data Availability

The datasets generated and/or analysed during the current study are available in the Open Science Framework repository, DOI 10.17605/OSF.IO/KQ4PM

## Abbreviations

AHN: Adult hippocampal neurogenesis
AN: Anorexia nervosa
ASD: Autism spectrum disorder
BDNF: Brain-derived neurotrophic factor
CA: Cornu ammonis
EDE-Q: Eating Disorder Examination Questionnaire
HC: Healthy control
HPA: Hypothalamic-pituitary-adrenal
LDI: Lure Discrimination Index
MAR: Missingness at random
MDD: Major depressive disorder
MNAR: Missingness not completely at random
MST: Mnemonic similarity task
SD: Standard deviation
SEM: Standard error of the mean
SSRIs: Selective serotonin reuptake inhibitors
STAI-T: State-Trait Anxiety Inventory – trait
