# Supplementary material for: Lower pattern recognition memory scores in anorexia nervosa
Source: J Eat Disord. 2021 Apr 17;9:49. doi: 10.1186/s40337-021-00406-8 (PMC8052530; doi:10.1186/s40337-021-00406-8)
Supplement: Supplementary file 1 — Additional file 1. [file 40337_2021_406_MOESM1_ESM.docx]

**Additional File 1**

| **Variable** | **N (%) missing** |
| --- | --- |
| Psychiatric comorbidities | 19 (18.4%) |
| Medication usage | 18 (17.5%) |
| Living status | 15 (14.6%) |
| EDE-Q Global Score | 14 (13.6%) |
| EDE-Q Weight Concern | 14 (13.6%) |
| EDE-Q Shape Concern | 14 (13.6%) |
| EDE-Q Eating Concern | 14 (13.6%) |
| EDE-Q Restraint | 14 (13.6%) |
| Trait anxiety (STAI-T) | 14 (13.6%) |
| Average sleep duration | 14 (13.6%) |
| Level of education | 13 (12.6%) |
| Relationship status | 13 (12.6%) |
| Ethnicity | 11 (11.7%) |

Supplementary Table 1. *Missing data rates for variables entered into the imputation model.*

*Note.* EDE-Q = Eating Disorder Examination Questionnaire; STAI-T = State-Trait Anxiety Inventory - Trait

|  | | **MST-O Task** | | | | | |
| --- | --- | --- | --- | --- | --- | --- | --- |
|  | | **Raw response rates** | | | **Corrected response rates** | | |
| **Group** | **Response** | **Repeat/Target** | **Lure** | **Foil** | **Repeat/Target** | **Lure** | **Foil** |
| **Healthy Controls**  **(*N*=56)** | **Old** | 0.75 (0.17) | 0.43 (0.13) | 0.07 (0.08) | 0.77 (0.17) | 0.44 (0.14) | 0.08 (0.09) |
|  | **Similar** | 0.14 (0.12) | 0.41 (0.16) | 0.13 (0.07) | 0.14 (0.12) | 0.42 (0.16) | 0.13 (0.08) |
|  | **New** | 0.09 (0.10) | 0.15 (0.10) | 0.78 (0.13) | 0.09 (0.11) | 0.15 (0.11) | 0.79 (0.12) |
| **Anorexia Nervosa**  **(*N*=46)** | **Old** | 0.77 (0.16) | 0.41 (0.13) | 0.06 (0.06) | 0.78 (0.17) | 0.42 (0.13) | 0.06 (0.06) |
|  | **Similar** | 0.14 (0.09) | 0.44 (0.15) | 0.13 (0.09) | 0.14 (0.09) | 0.45 (0.15) | 0.13 (0.10) |
|  | **New** | 0.08 (0.12) | 0.12 (0.14) | 0.78 (0.15) | 0.08 (0.14) | 0.13 (0.15) | 0.81 (0.14) |

Supplementary Table 2. *Response proportions and standard deviations for each stimulus and response type per group.*

|  | Pattern Separation Score  (N=34) | Recognition Memory Score (N=34) | Age (N=34) | Body Mass Index  (N=31) | Eating Disorder Symptoms  (N=34) | Trait Anxiety  (N=34) |
| --- | --- | --- | --- | --- | --- | --- |
| Pattern Separation Score | 1 |  |  |  |  |  |
| Recognition Memory Score | 0.39* | 1 |  |  |  |  |
| Age | 0.02 | -0.12 | 1 |  |  |  |
| Body Mass Index | 0.04 | -0.09 | 0.06 | 1 |  |  |
| Eating Disorder Symptoms | 0.23 | -0.17 | 0.06 | 0.09 | 1 |  |
| Trait Anxiety | 0.13 | -0.10 | 0.07 | 0.05 | 0.37 | 1 |
| *Note.* Significantly correlated at: *p<0.05, (2 tailed). | | | | | | |

Supplementary Table 3. *Bivariate correlation matrix between pooled demographic variables and MST outcomes for the clinical group*

Supplementary Table 4. *Bivariate correlation matrix between pooled demographic variables and MST outcomes for the control group*

|  | Pattern Separation Score  (N=56) | Recognition Memory Score (N=40) | Age  (N=56) | Body Mass Index  (N=56) | Eating Disorder Symptoms  (N=56) | Trait Anxiety  (N=56) |
| --- | --- | --- | --- | --- | --- | --- |
| Pattern Separation Score | 1 |  |  |  |  |  |
| Recognition Memory Score | 0.18 | 1 |  |  |  |  |
| Age | -0.18 | -0.05 | 1 |  |  |  |
| Body Mass Index | -0.09 | -0.24 | 0.36* | 1 |  |  |
| Eating Disorder Symptoms | <0.01 | <-0.01 | -0.10 | 0.28 | 1 |  |
| Trait Anxiety | 0.07 | 0.06 | -0.02 | 0.09 | 0.50* | 1 |
| *Note.* Significantly correlated at: *p<0.05, (2 tailed). | | | | | | |
